# Overuse of CT for Minor Head Trauma Patients: A Retrospective Analysis from Poland and Lithuania Trauma Centres

**DOI:** 10.3390/medicina60121908

**Published:** 2024-11-21

**Authors:** Kristina Blažienė, Jakub Nożewski, Vaida Cibulskė, Monika Kunigonytė, Deimantė Košytė, Karolis Bareikis, Vytautas Aukštakalnis

**Affiliations:** 1 Emergency Medicine Department, Lithuanian University of Health Sciences, 44307 Kaunas, Lithuania; vaida.vrubliauskaite@stud.lsmu.lt (V.C.); monika.kunigonyte@stud.lsmu.lt (M.K.);; 2 Emergency Medicine Department, Jan Biziel University Hospital no 2, 85-168 Bydgoszcz, Poland; jbnoz@wp.pl; 3 Department of Neurosurgery, Lithuanian University of Health Sciences, 44307 Kaunas, Lithuania

**Keywords:** minor head trauma, overuse of head CT, minor head injury

## Abstract

*Background and Objectives*: Head trauma is one of many conditions that trauma centres deal with daily. This study aimed to analyse the utilisation of head CT scans for patients with minor head trauma in two major hospitals in Lithuania and Poland. *Materials and Methods*: We conducted a retrospective, descriptive study of CT utilisation in minor head trauma patients presenting to the Level 1 trauma centre Hospital of the Lithuanian University of Health Sciences Kaunas Clinics (HLUHS KC) and Jan Biziel University Hospital in Bydgoszcz emergency departments (EDs), during the study period from 01 February to 30 April 2023. *Results*: During the study period, 1048 patients visited the HLUHS KC emergency department (ED) due to head trauma, and 388 patients visited the Jan Biziel University Hospital. Overall, 611 patients were included in the study. Most of the patients (92%) who suffered minimal trauma were younger than 65 years old. Eighty-two per cent of the patients older than 65 years old arrived at the ED after suffering a fall. Almost all the patients who were using antiplatelets (93%) or anticoagulants (91%) had CT scans. Non-emergency medicine (EM) physicians were more likely to order head CT scans than EM physicians (170 (83%) vs. 249 (62%), *p* < 0.001). There were 33 (5%) CT scans with traumatic features, and 8 (1%) of these were categorised as clinically significant. Patients who suffered clinically significant head trauma were more likely to be on anticoagulants and older than 65 when compared to normal/insignificant CT findings: 3 (38%) vs. 25 (6%), *p* < 0.001; and 6 (75%) vs. 146 (36%), *p* < 0.021. *Conclusions*: A significant number of head CT scans performed were not necessary according to existing head CT guidelines and risk calculators. However, even in minor head traumas, clinically significant head injuries may occur.

## 1. Introduction

Head trauma is one of many conditions that trauma centres deal with daily. Based on data from 2019, around 4.67 million people in Europe suffered a head injury, with most cases being from Western Europe [1]. In Lithuania in 2022, 57,801 patients suffered a head trauma, of which 4298 (7.44%) were hospitalised. Among both outpatients and inpatients, males were more likely to have sustained injuries than females. The most common cause of head trauma by mechanism of injury was falling [2].

There are no official data on the quantity of head computed tomography (CT) scans performed for patients with minor head trauma in Lithuania. Based on our calculations from the Hospital of Lithuanian University of Health Sciences Kaunas Clinics (HLUHS KC), approximately 1700 patients with minor head trauma receive head CT scans every year. This costs roughly EUR 121,000 yearly from the national healthcare budget. Since there are three third-level, four second-level and six first-level trauma centres in Lithuania, these expenses are probably at least several times higher. In Jan Biziel University Hospital, around 980 patients receive head CT scans after minor head trauma each year, costing around EUR 65,000 per year.

Along with the cost of a CT scan, patients who undergo scanning also must wait for test results, which can significantly extend their stay in the emergency department (ED), resulting in another cause of bottlenecks [3].

CT exposes a patient to radiation, and a non-contrast head CT scan delivers a dose of approximately 2 mSv effective radiation. This dose equals approximately eight months of natural background radiation [4]. Every computed tomography scan is thought to increase the risk of cancer due to radiation. However, the calculations are very complicated, and the risk of developing cancer after a single test is unclear [5].

Evidence-based medicine and risk calculators, such as the New Orleans/Charity Head Trauma/Injury Rule, Canadian Head CT Rule, and NEXUS Head CT decision instrument, enable the clear identification of patients who require CT. Research shows that in head injuries alone, one in three CT scans are performed without any indications [6].

Either way, patients with minor head trauma can have intracranial bleeding or other life-threatening findings, depending on risk factors and the mechanism of trauma. These findings can be significant and require hospitalisation or even surgical treatment [7,8]. Patients with traumatic brain injuries can also have long-term consequences on their physical and psychological health, which can lead to decreased working ability, increased medical expenses and a burden on our healthcare system [9,10,11,12].

This study aimed to analyse the utilisation of head CT scans for patients with minor head trauma in two major hospitals in Lithuania and Poland.

## 2. Materials and Methods

We conducted a retrospective, descriptive study of minor head trauma patients presenting to the Level 1 trauma centre hospital of the HLUHS KC and Jan Biziel University Hospital in Bydgoszcz emergency departments (EDs) from 01 February to 30 April 2023. Inclusion criteria were all adult (≥18 years old) patients presenting to the ED within 48 h after minor head trauma. Exclusion criteria included a documented loss of consciousness or unknown, Glasgow Coma Scale (GCS) < 15 or any neurological deficit, high-energy trauma (e.g., pedestrian struck by motor vehicle, occupant ejected from a motor vehicle, or a fall from more than three feet or five stairs), suspected cranial vault or skull base fracture, and penetrating head injury.

All patients’ medical charts with final diagnoses coded in ICD-10 S00, S01, S02 and S06 were reviewed. Only the data of patients meeting the inclusion criteria were included in the final analysis. Variables analysed were age, gender, mechanism of injury, use of anticoagulants and antiplatelets, CT findings, whether the patient arrived by EMS, visible head laceration, need for surgery, length of stay, survival status and return to hospital due to head trauma symptoms within six weeks. All the CT scans were interpreted by the radiologist during the ED stay. CT scans with acute traumatic findings were retrospectively reviewed by a neurosurgeon and categorised as clinically significant or insignificant. The significance of intracranial findings was evaluated based on whether they could account for neurological symptoms, necessitate surgical intervention, or require follow-up due to potential expansion with subsequent need for surgical or intensive care management. Criteria assessed on CT scans included the presence of displaced cranial vault fractures, skull base fractures, brain contusions or intracerebral haemorrhages, extensive subarachnoid haemorrhage involving two or more lobes and/or the basal cisterns, and epidural or subdural haemorrhages of any diameter.

All data were analysed using descriptive analysis with IBM^®^ SPSS^®^ statistics, version 21 (IBM Corp., Armonk, NY, USA). Qualitative data are presented as absolute numbers (n) and percentage (%). Quantitative data are presented as median with interquartile ranges (Q1–Q3).

We conducted a subgroup analysis focusing on patients who, based on the Canadian Head CT Rule and NEXUS Head CT decision instrument, were deemed ineligible for head CT scans.

To determine whether the data were normally distributed, we performed a Shapiro–Wilk normality test. Since all the data were not normally distributed, we used non-parametric Mann−Whitney U-test. Categorical variables were compared using Pearson’s Chi-square test of proportion; in categories with *n* < 5, Fisher’s exact test was used.

A difference was considered statistically significant if *p* < 0.05. For missing data, the last observation carried forward method was used.

## 3. Results

During the study period, 1048 patients visited the HLUHS KC ED due to head trauma, and 388 patients visited the Jan Biziel University Hospital. Overall, 611 patients met the inclusion criteria and were included in the study. In our study population, according to Canadian Head CT Rule or NEXUS Head CT decision instrument 174 (28.5%) head CT scans should have been considered or performed due to age or the use of blood thinners. However, 419 (69%) of the study population underwent CT scans.

The median age of the patient group for whom CT scans were performed was higher than those who did not receive a CT scan: 53 years (range: 34–73) compared to 32 years (range: 25–43), respectively, *p* < 0.001. CT was performed for 226 (67%) males and 193 (72%) females (*p* = 0.23). Patients who arrived by EMS were more likely to receive a CT scan (265 (87%) vs. 156 (51%), *p* < 0.001). Trauma mechanisms are shown in Table 1.

Most of the patients (92%) who suffered minimal trauma were younger than 65. Eighty-two per cent of the patients older than 65 years old arrived at the ED after suffering a fall. Almost all the patients who were using antiplatelets or anticoagulants had a CT scan (25 (93%) and 28 (91%), respectively). Patients who suffered head lacerations were less likely to be CT scanned (150 (64%) vs. 269 (72%), *p* = 0.046). Non-emergency medicine (EM) physicians were more likely to order head CT scans than EM physicians (170 (83%) vs. 249 (62%), *p* < 0.001).

There were 33 (8%) abnormal CT scans, and 8 (1%) of these were categorised as clinically significant (Table 2). Patients who suffered clinically significant head trauma were more likely to be on anticoagulants and older than 65 compared to normal/insignificant CT findings: 3 (38%) vs. 25 (6%), *p* < 0.001; and 6 (75%) vs. 146 (36%), *p* < 0.021. None of the patients received a specific interventional treatment.

None of the patients in the study population died or revisited the hospital within a six-week period for symptoms related to head trauma. All the patients survived to discharge.

### Subgroup Analysis

There were 437 (72%) patients for whom head CT scans should not have been performed. Two hundred and sixty-four (60%) patients were scanned, and 24 scans were found to be abnormal; however, only two patients had significant findings (Table 2, patients 1 and 2). Non-EM physicians were more likely to order head CT scans than EM physicians (109 (77%) vs. 152 (51%), *p* < 0.001).

## 4. Discussion

In this study, we analysed the utilisation of head CT scans for patients with minor head trauma in Lithuania and Poland hospitals. Despite the existence of several validated calculators and criteria for over two decades, the number of head CT scans performed for mild head trauma remains high in Lithuania and Poland. In our study population subgroup analysis, 264 CT scans were deemed unnecessary based on the Canadian Head CT Rule and NEXUS Head CT decision instrument. Based on our extrapolated data and calculations, avoiding unnecessary scans would have saved almost EUR 12,000 over three months, which could be extrapolated to EUR 48,000 per year. And it would be hard to say the exact number if we could multiply it by all the hospitals that are performing head CT scans in Poland and Lithuania.

Our study found that head CT is more frequently performed in older people admitted to the ED. Most previous studies have reported that older patients are more likely to undergo a head CT scan following mild head trauma. This inclination can be attributed to physiological changes associated with ageing, comorbidities and the use of anticoagulants, all of which are linked to a higher incidence of intracranial haemorrhage [13]. In our study, we also observed that patients over 65 were independently more likely to undergo a head CT. Furthermore, 75% of patients diagnosed with clinically significant intracranial haemorrhages were within this age group.

When analysing trauma mechanisms, we found that falling was the most common mechanism resulting in a CT scan. Within this group, most patients were older than 65. These findings are consistent with the conclusions of other recent studies [14,15,16]. It is not surprising that the most common trauma mechanism in the group not undergoing CT scans was minimal trauma among younger patients. It is highly unlikely to have a clinically significant intracranial injury in youth from simply bumping their head on a glass door or during a basketball game, or from any other minimal trauma.

We found that almost all patients presenting to the ED with minimal head trauma and taking anticoagulants underwent scanning. Of these patients, 11% had clinically significant findings in their CT scans. These findings align with previous research, where intracranial haemorrhages ranged from 2% to 16% [13,14,15,17,18,19,20].

An interesting result is that patients who were found to have a head wound were scanned less frequently. This could be attributed to the anchoring effect, whereby a physician, upon observing a major external injury, may overlook important details during the history-taking or physical examination that could warrant scanning prior to suturing. It is worth noting that Forouzannia et al. reported that a visible injury above the clavicle is associated with abnormal head CT findings [21]. However, in our study, none of the patients returned to the emergency department due to missed diagnoses.

Our results show that only a very small number of patients with minimal head trauma have abnormalities on head CT. After the review of positive CT scans by an expert neurosurgeon, only eight (1.3%) clinically significant changes were identified. None of the patients required urgent neurosurgical intervention. This correlates with studies in other countries with similar results of abnormal head CT 1–4% [16,19,22]. However, in other studies, concerning results were observed, showing abnormal CT results up to 5–12%, with neurosurgical intervention between 1 and 3% and death rates ranging from 0.1 to 0.9% [15,22,23,24].

The EM residency was introduced in Lithuania in 2013, leading to the staffing of the ED not only by EM physicians but also by specialists from other fields. In our study, we observed that EM physicians performed fewer unnecessary CT scans. However, the literature presents mixed results regarding whether emergency medicine physicians perform fewer unnecessary scans [22,23,25]. One suggestion to explain this result in our hospitals is that despite being a young speciality, EM doctors possess broad expertise, enabling them to assess trauma and neurological examinations using an evidence-based approach. They employ risk stratification tools rather than relying solely on clinical experience or dogma.

Based on the work by Nożewski J et al. and an analysis of the topic, we believe a significant problem in Poland among physicians is the fear of medical error and litigation [26]. This issue is exacerbated by the predominant use of contractual employment, where full liability rests on the individual physician. Defensive medicine is a global issue; physicians do not consider the risks of radiation exposure or the costs associated with unnecessary diagnostic tests. This leads to absurd situations where even the implementation of hospital guidelines or references to widely used clinical calculators fail to reduce the number of CT scans.

We believe that defensive medicine is of great importance in Lithuania. In our opinion, when doctors with more clinical experience tend to remember the worst cases, they perform more CT scans in order to avoid possible poor outcomes for the patient. We also think that, on the contrary, doctors who do not yet have as much clinical experience, especially in working with head injuries, order more CT examinations due to the fear of making a mistake or not making the correct diagnosis.

Our study has several limitations. First, our study was a retrospective chart review study. Second, this study was conducted in two hospitals for only 3 months, which may not represent the entire patient population. Third, we did not follow up with the included patients, and it was not possible to know if they had lethal outcomes at home within the study period. Also, due to poor documentation, it was not clear if the patients received any specific medical treatment during the hospitalisation period. We aimed to analyse CT utilisation in patients with very minor traumas. However, the lack of standardised patient inclusion criteria limits the comparability of our findings with other studies.

## 5. Conclusions

Our study highlights the significant overuse of head CT scans for minor head trauma patients in Lithuanian and Polish hospitals. Despite established guidelines and risk calculators, a substantial proportion of scans were deemed unnecessary. This overuse leads to increased healthcare costs and unnecessary radiation exposure for patients.

While a very small percentage of patients with minor head trauma had clinically significant findings on CT scans, these were primarily minor haemorrhages requiring no surgical intervention.

## Figures and Tables

**Table 1:** Mechanisms of trauma.

Mechanism	CT Performed, *n* (%)	No CT, *n* (%)	*p*
Car crash	25 (6)	11 (6)	0.95
Bicycle accident	7 (2)	0	0.11
Scooter accident	5 (1)	3 (2)	0.71
Beaten	64 (15)	28 (15)	0.83
Minimal trauma	32 (8)	75 (40)	<0.001
Struck by object	30 (7)	8 (4)	0.17
Fall	239 (57)	58 (31)	<0.001
Other	16 (4)	7 (4)	0.95

**Table 2:** Patients with clinically significant CT findings.

Patient	Centre	Age	Gender	History	Trauma Mechanism	CT Finding	Length of Stay	Anticoagulant
1	LT	62	Female	Fell onto the back of the head after being struck by a bicyclist. Felt dizzy, was hard to stand up.	Bicycle	SAH ^1^	5	No
2	LT	56	Male	Punch to the nose, small nose laceration.	Beaten	SDH ^2^	2	No
3	LT	78	Female	Dizziness was the reason for the fall on the hard surface. Laceration in temporal area.	Fall	SAH ^1^	13	Edoxaban
4	PL	76	Female	Slipped and fell on the back of the head.	Fall	SAH ^1^	2	No
5	LT	98	Female	Tripped and fell several times, feels dizzy.	Fall	SDH ^2^	4	No
6	LT	68	Female	Slipped and fell on the back of the head twice. Woke up in the morning nauseous and dizzy.	Fall	ICH ^3^	8	Rivaroxaban
7	PL	77	Female	Slipped, wound on the forehead. Headache and nausea.	Fall	SDH ^2^	2	Warfarin
8	PL	79	Female	Head trauma, nose fracture, zygomatic trauma, nosebleeds, ocular hematomas.	Fall	ICH ^3^	1	No

^1^ SAH: subarachnoid haemorrhage; ^2^ SDH: subdural haemorrhage; ^3^ ICH: intracerebral haemorrhage.

## Data Availability

The original contributions presented in this study are included in the article; further inquiries can be directed to the corresponding authors.
